# Economic evaluation of patient costs associated with tuberculosis diagnosis and care in Solomon Islands

**DOI:** 10.1186/s12889-021-11938-8

**Published:** 2021-10-23

**Authors:** Kerri Viney, Noel Itogo, Takuya Yamanaka, Ridha Jebeniani, Anupama Hazarika, Fukushi Morishita, Nobuyuki Nishikiori, Susana Vaz Nery

**Affiliations:** 1 Research School of Population Health, Australian National University, Canberra, Australia; 2 Global TB Programme, World Health Organization Headquarters, Geneva, Switzerland; 3 Department of Global Public Health, Karolinska Institutet, Stockholm, Sweden; 4 National TB Programme, Ministry of Health and Medical Services, Honiara, Solomon Islands; 5 World Health Organization Country Office, Honiara, Solomon Islands; 6 Department of Global Health and Development, London School of Hygiene & Tropical Medicine, London, UK; 7 School of Tropical Medicine & Global Health, Nagasaki University, Nagasaki, Japan; 8 Independent consultant, Tunis, Tunisia; 9 End TB and Leprosy Unit, World Health Organization Regional Office of the Western Pacific, Manila, Philippines; 10 The Kirby Institute, University of New South Wales Sydney, Sydney, Australia

**Keywords:** Tuberculosis, Costs, Solomon Islands, Economic impact, Health care access

## Abstract

**Background:**

Tuberculosis (TB) care can be costly for patients and their families. The End TB Strategy includes a target that zero TB affected households should experience catastrophic costs associated with TB care. Costs are catastrophic when a patient spends 20% or more of their annual household income on their TB diagnosis and care. In Solomon Islands the costs of TB care are unknown. The aim of this study was to determine the costs of TB diagnosis and care, the types of costs and the proportion of patients with catastrophic costs.

**Methods:**

This was a nationally representative cross-sectional survey of TB patients carried out between 2017 and 2019. Patients were recruited from health care facilities, from all ten provinces in Solomon Islands. During an interview they were asked about the costs of TB diagnosis and care. These data were analysed using descriptive statistics to describe the costs overall and the proportions of different types of costs. The proportion of patients with catastrophic costs was calculated and a multivariate logistic regression was undertaken to determine factors associated with catastrophic costs.

**Results:**

One hundred and eighty-three TB patients participated in the survey. They spent a mean of 716 USD (inter quartile range: 348–1217 USD) on their TB diagnosis and care. Overall, 62.1% of costs were attributable to non-medical costs, while income loss and medical costs comprised 28.5 and 9.4%, respectively. Overall, 19.7% (*n* = 36) of patients used savings, borrowed money, or sold assets as a financial coping mechanism. Three patients (1.6%) had health insurance. A total of 92.3% (95% CI: 88.5–96.2) experienced catastrophic costs, using the output approach. Being in the first, second or third poorest wealth quintile was significantly associated with catastrophic costs (adjusted odds ratio: 67.3, 95% CI: 15.86–489.74%, *p* <  0.001).

**Conclusion:**

The costs of TB care are catastrophic for almost all patients in Solomon Islands. The provision of TB specific social and financial protection measures from the National TB and Leprosy Programme may be needed in the short term to ameliorate these costs. In the longer term, advancement of universal health coverage and other social and financial protection measures should be pursued.

**Supplementary Information:**

The online version contains supplementary material available at 10.1186/s12889-021-11938-8.

## Background

The costs of tuberculosis (TB) diagnosis and care are thought to be a significant impediment to accessing health care for patients, and to attaining national and global targets of reductions in TB incidence and mortality for national TB programmes [1]. Previous studies have documented that TB patients often incur large costs related to their illness, as well as seeking and receiving health care [2, 3]. Such costs can create barriers to health care access and treatment adherence, which can affect health outcomes and increase the risk of TB transmission. These costs can also be detrimental to the economic situation of households as TB predominantly affects people of working age. By way of example, in low-and middle-income countries, TB patients face costs that on average amount to half of their annual income [2]. Lost income is often the dominant contributor to economic hardship; however out-of-pocket medical expenditures are also important [4–6]. In addition, costs for travel, food and relocation during health seeking are also significant, given the often long health seeking period and the six-month to two-year period of TB treatment that is the internationally accepted standard for treatment to be effective [2]. In fact, a previous systematic review determined that these costs are equivalent to 58% of annual individual income and 39% of annual household income, worse for people who were already poor or who had multidrug-resistant (MDR)-TB [2].

The costs of TB diagnosis and care can be estimated through national TB patient cost surveys using a methodology designed by the World Health Organization (WHO) [7]. Using this method, measurement of a range of patient costs due to TB help to identify areas for improvement in TB service delivery and financing, and contribute to the measurement of the *End TB Strategy* indicator for the target that zero TB affected households should experience catastrophic costs related to TB, to be attained by the year 2020 [1]. Costs are said to be “catastrophic” when spending on TB is equal to or greater than 20% of the TB patients’ annual household income [1]. The results of such surveys are important to assess the magnitude of patient costs and identify the main categories of costs, which can then be used to monitor financial barriers to accessing and adhering to TB services and inform related health and social policy changes to improve TB care [8].

Solomon Islands is a medium burden TB country. It reports approximately 400 TB case notifications per year, resulting in a TB case notification rate of 62 cases per 100,000 population [9, 10]. A joint programme review of the National TB and Leprosy Programme (NTLP) in 2019 identified multiple barriers for patients in accessing TB services along their care pathways. TB patients often present late for a TB diagnosis, mainly due to a lack of awareness about TB, and difficult access to health facilities due to the country’s geography and lack of public transport [11]. Health centres also lack laboratory and human resource capacity to diagnose and initiate TB treatment, so they often refer patients to a provincial hospital [11]. If the patient is diagnosed with TB, historically they have been hospitalized for two months and then after this, they are discharged and receive treatment closer to their home [11]. However, the real costs of TB care are unknown in Solomon Islands.

Therefore, this study aimed to conduct a baseline assessment of the economic burden of TB on patients and their households in Solomon Islands, using the WHO recommended national TB patient survey methodology, something that was also recommended during the 2019 programme review [11]. The specific aims were to determine the financial burden of TB for patients in Solomon Islands and to inform policies to mitigate costs and improve access to TB care.

## Methods

### Setting

Solomon Islands is comprised of over 900 islands and is located in the Pacific Ocean [12]. It has a population of approximately 640,000 people, of whom 80% live in rural areas [12, 13]. It is a lower middle income country [9] where 12.7% of the population live below the national poverty line [14]. Health care in Solomon Islands is largely provided by the public sector and out-of-pocket spending on health is low [15]. However, non-medical expenditures, particularly for transportation to access health care, are reported to be high [16, 17]. The Solomon Islands NTLP is based in Honiara (the capital). Then, in all ten provinces, there is a designated health facility or hospital that serves as a TB Directly Observed Treatment Short-course (DOTS) centre, which is staffed by a Provincial Tuberculosis and Leprosy Coordinator. It provides TB services and serves as a TB Basic Management Unit for that province. The country has been implementing the World Health Organization (WHO) recommended strategy for controlling TB, which evolved from the DOTS strategy, the Stop TB strategy and currently the End TB Strategy. For the past decades, the NTLP has made good progress against a number of programmatic indicators [11]. For example, the treatment coverage and treatment success rates in 2017 were 80 and 92% respectively and the proportion of TB patients who were bacteriologically confirmed in 2018 was 65% [11]. However, various challenges remain; including; access barriers, limited implementation planning across all levels of the health system, resource constraints which limit outreach services and inconsistent use of preventive therapy in eligible risk groups [11].

### Study design

This was cross-sectional survey of TB patients including all ten DOTS centres offering TB treatment in the country, and therefore a nationally representative study that aimed to assess the costs associated with TB diagnosis and care.

### Sampling approach and study population

The sample size was estimated at 228 patients. This figure was based on the number of DOTS centres offering TB treatment (*n* = 10), and the number of patients diagnosed in Solomon Islands per year (at the time of protocol development in 2016, this was *n* = 360), assuming a level of 30% catastrophic costs and a 5% non-participation rate. At the time, 30% was deemed a reasonable estimate given the pre existing knowledge on this topic [2]. The study population consisted of all diagnosed TB patients (of all ages and with all types of TB) who had received TB treatment for at least two weeks whether they were in the intensive phase or the continuation phase, from the selected DOTS centres.

### Questionnaire

The WHO have developed a standardised, structured questionnaire for TB patient cost surveys which was adapted to the Solomon Islands context (Additional file 1). The questionnaire contained information on clinical parameters; demographic variables and household composition; socioeconomic variables; health care utilisation; time spent seeking and receiving care; direct medical, direct non-medical and indirect costs; household and individual income; household assets; financial and social coping mechanisms and the perceived impacts of costs. The questionnaire contained closed and open-ended questions. It was pilot tested and further refined after a two-day national training on data collection methods and interview techniques. The questionnaire was uploaded into Open Data Kit collect within the Ona platform (https://ona.io/home/), and was designed with built-in options and skips to promote accurate data collection.

### Patient interviews

Interviewers were trained Provincial TB and Leprosy Co-ordinators or other NTLP staff (i.e. health care workers). They approached TB patients to be involved in the study as they attended clinic visits. Interviews took place at the clinic or during follow up home visits in a separate space or outside, to allow for privacy and to ensure optimal infection control practices. All patients interviewed were on effective treatment for more than two weeks prior to the interview, and as such the infection risk was minimal. Interviewers read the questions to TB patients in Solomon Islands Pigin (the lingua franca in Solomon Islands), and recorded responses directly into a tablet. For children aged under 18 years, an accompanying parent or guardian answered the questions. The interviews took 60–90 min to complete.

### Data collection and management

Data were collected between 2017 and 2019. Once the interview was finished, interviewers submitted the data in the Ona platform; data were then sent directly to a password protected server, also in Ona. A small number of questionnaires were completed on a paper version (a direct replication of the electronic version); these were then sent to the NTLP Co-ordinator who entered them into Ona. During the period of data collection, remote checks of the data were undertaken by the survey investigators; any obvious or repetitive errors were fed back to the NTLP Co-ordinator and the interviewers. Then, in December 2019, during a Provincial TB and Leprosy Co-ordinator training, each record was checked by an experienced external consultant and any errors were corrected (T Yamanaka, personal communication, July 2020).

### Definitions and data analysis

Direct medical costs were defined as “out-of-pocket payments made by TB-affected patients or their guardians for medical services (i.e. consultations, tests, medicines, other medical procedures), net of any reimbursements” while direct non-medical costs were defined as “out-of-pocket payments made by TB-affected patients or guardians related to transportation, accommodation, food, nutritional supplements”, net of any reimbursements [7]. Indirect costs were “productivity and economic costs of a TB patient or someone in their household incurred as a result of TB health care visits and hospitalization during the TB episode.” [7]. Catastrophic costs were defined as the sum of direct medical, direct non-medical and indirect costs, net of any welfare payment that exceeded 20% of the household’s income [1].

Annual household income was estimated using two approaches. An adjusted approach was added as Solomon Islands has a large informal and cash based economy [7]. Using the output approach, indirect costs were measured using “self-reported household income at three points in time (prior to the onset of TB symptoms, at the time of diagnosis and during the current treatment phase) to estimate income change before and during the TB episode.” [7]. For households without income information, it was imputed using a regression model based on household assets information. Using a second (adjusted) approach, household income was adjusted to account for the proportion of the population with non-cash based income, using the results from the most recent Solomon Islands Household Income Expenditure Survey (HIES); completed in 2013 [18]. The results of the HIES revealed that cash-based income accounted for 63% of total annual income, and this proportion was considerably lower in rural areas and for poor households [18]. Self-reported household income was then adjusted using the following method:

1. the reported household income was assumed to be “cash-based” income;
2. for those who did not have household income information, it was imputed based on household assets;
3. HIES income decile groups were applied to reported household income (Supplementary Table 1);
4. household cash-based income was divided by the proportion of households with cash- based income observed in the HIES (by decile).

All analyses were carried out using the statistical software package R 4.0.2 (CRAN:Comprehensive RArchive Network at https://cran.r-project.org/). R scripts prepared by the Global TB Programme, WHO, were used as the basis for analyses with modifications for the analytical approach. The study population was described using descriptive statistics with mean or median values used as appropriate. Median medical, non-medical costs and indirect costs, before TB treatment started and during TB treatment were calculated. Medical, non-medical and indirect costs as a proportion of all costs were also determined, as was the proportion of TB patients who incurred catastrophic costs. To estimate the costs of TB care for the entire period of TB diagnosis and care, we imputed costs based on data from patients in a certain treatment phase (i.e. intensive or continuation phase). This is the approach recommended by WHO, where missing cost data for a particular phase of TB treatment are replaced by the median values obtained from other survey participants in the same phase. Patients with pulmonary TB and extra-pulmonary TB were compared using the chi-square test for categorical data and the Welsh T-test or two-sample Wilcoxon rank-sum test for continuous data. Finally, a stepwise multivariate regression analysis was undertaken to determine which binary variables were independently associated with catastrophic costs. We used the adjusted household income for the regression analysis, as the proportion of patients with catastrophic costs was lower (i.e. it was more conservative). Adjusted odds ratios with their associated 95% confidence intervals (CIs) and *p* values were presented. Measures of association were considered significant at the 5% significance level.

## Results

### Demographic and clinical characteristics

A total of 183 TB patients participated in the survey; 147 (80.3%) had pulmonary TB (PTB) and 36 (19.7%) had extra-pulmonary TB (EPTB) (Table 1). There were no statistically significant differences between patients with EPTB and PTB except for the year of TB registration and province of residence (*p* = 0.036 and 0.028, respectively). Ninety-two (50.3%) patients were male and the mean age was 32.8 years (95% CI: 30.3–35.2). Only 23 (12.6%) reported being employed before their TB diagnosis and overall, the median monthly household income was USD 22.2 (inter-quartile range (IQR) 12.3–91.1) or 180.0 Solomon Island dollars (IQR 100.0–740.0)). All patients were being treated for drug susceptible TB, 97 (53.0%) were in the intensive phase, and 95 (51.9%) were hospitalized at the time of interview (Table 2). The only statistically significant differences when comparing the clinical characteristics of patients with EPTB and PTB were the proportion of patients who were bacteriologically confirmed (13.9% vs 78.9%, *p* <  0.001, for EPTB and PTB respectively) and the mean duration of the intensive phase (2.3 months vs. 2.1 months, *p* = 0.048, for EPTB and PTB, respectively). Fifty-three percent (*n* = 44) patients reported a diagnostic delay (defined as an interval between onset of symptoms and a TB diagnosis of four weeks or more) (Supplementary Table 2).

**Table 1:** Socio-demographic characteristics of patients who participated in the Solomon Islands national tuberculosis patient cost survey: 2017–2019

Socio-demographic characteristics	Extra-pulmonary TB *N* = 36 n (%)	Pulmonary TB *N* = 147 n (%)	All patients *N* = 183 n (%)	*p* value*
Age group (years)				
0–14	3 (8.3)	18 (12.2)	21 (11.5)	0.964
15–24	10 (27.8)	31 (21.1)	41 (22.4)	
25–34	8 (22.2)	32 (21.8)	40 (21.9)	
35–44	8 (22.2)	30 (20.4)	38 (20.8)	
45–54	4 (11.1)	20 (13.6)	24 (13.1)	
55–64	2 (5.6)	9 (6.1)	11 (6.0)	
65+	1 (2.8)	7 (4.8)	8 (4.4)	
Mean (95% CI)	31.8 (26.4, 37.2)	33.0 (30.2, 35.8)	32.8 (30.3, 35.2)	0.700
Sex				
Female	19 (52.8)	72 (49.0)	91 (49.7)	0.683
Male	17 (47.2)	75 (51.0)	92 (50.3)	
Year of TB registration				
2017	0 (0.0)	13 (8.8)	13 (7.1)	0.036
2018	20 (55.6)	95 (64.6)	115 (62.8)	
2019	16 (44.4)	39 (26.5)	55 (30.1)	
Province				
Central	1 (2.8)	4 (2.7)	5 (2.7)	0.028
Choiseul	0 (0.0)	5 (3.4)	5 (2.7)	
Guadalcanal	7 (19.4)	15 (10.2)	22 (12.0)	
Honiara City	16 (44.4)	35 (23.8)	51 (27.9)	
Makira	2 (5.6)	21 (14.3)	23 (12.6)	
Malaita	10 (27.8)	36 (24.5)	46 (25.1)	
Temotu	0 (0.0)	11 (7.5)	11 (6.0)	
Western	0 (0.0)	19 (12.9)	19 (10.4)	
Ysabel	0 (0.0)	1 (0.7)	1 (0.5)	
Insurance status				
No insurance	36 (100.0)	144 (98.0)	180 (98.4)	0.387
With insurance	0 (0.0)	3 (2.0)	3 (1.6)	
Education level				
No education	9 (25.7)	23 (15.6)	32 (17.6)	0.560
Pre- or primary school	16 (45.7)	75 (51.0)	91 (50.0)	
Secondary school	8 (22.9)	41 (27.9)	49 (26.9)	
University, vocational or other	2 (5.7)	8 (5.4)	10 (5.5)	
Employment status, pre-disease				
Employed	7 (19.4)	16 (10.9)	23 (12.6)	0.265
Unemployed	14 (38.9)	75 (51.0)	89 (48.6)	
Student, retired, household work or other	15 (41.7)	56 (38.1)	71 (38.8)	
Household size				
Mean (95% CI)	6.7 (5.7, 7.7)	6.7 (6.3, 7.2)	6.7 (6.3, 7.1)	0.970
Reported household income, monthly, pre-disease, monthly				
Median (IQR)	12.3 (0.0, 87.4)	24.6 (12.3, 92.4)	22.2 (12.3, 91.1)	0.252
TB patient was main income earner				
Yes	1 (2.8)	8 (5.4)	9 (4.9)	0.694
No	14 (38.9)	66 (44.9)	80 (43.7)	
Equal contributor	13 (36.1)	40 (27.2)	53 (29.0)	
Not an income earner	8 (22.2)	33 (22.4)	41 (22.4)	

*Abbreviations*: *CI* Confidence interval; *IQR* Inter quartile range; *TB* Tuberculosis.* *P* values compare the proportions of patients with the characteristic of interest, in the pulmonary and extra-pulmonary groups

**Table 2:** Clinical information of patients who participated in the Solomon Islands national tuberculosis patient cost survey: 2017–2019

Clinical characteristics	Extra-pulmonary TB ***N*** = 36 n (%)	Pulmonary TB ***N*** = 147 n (%)	All patients ***N*** = 183 n (%)	***p*** value*
**Treatment registration group**				
New	34 (94.4)	136 (92.5)	170 (92.9)	0.917
Relapse	1 (2.8)	5 (3.4)	6 (3.3)	
Retreatment excluding relapse	1 (2.8)	6 (4.1)	7 (3.8)	
**Drug resistance status**				
Drug susceptible-TB	36 (100.0)	147 (100.0)	183 (100.0)	–
**HIV status**				
Negative	24 (66.7)	92 (62.6)	116 (63.4)	0.813
Positive	0 (0.0)	1 (0.7)	1 (0.5)	
Unknown	12 (33.3)	54 (36.7)	66 (36.1)	
**Drug susceptibility testing conducted**				
DST done	0 (0.0)	2 (1.4)	2 (1.1)	0.482
DST not done	36 (100.0)	145 (98.6)	181 (98.9)	
**Mode of TB diagnosis**				
Bacteriologically confirmed	5 (13.9)	116 (78.9)	121 (66.1)	**< 0.001**
Clinically diagnosed	31 (86.1)	31 (21.1)	62 (33.9)	
**Treatment phase**				
Intensive phase	21 (58.3)	76 (51.7)	97 (53.0)	0.475
Continuation phase	15 (41.7)	71 (48.3)	86 (47.0)	
**Duration of intensive phase (months, mean (95% CI))**	2.3 (2.0, 2.5)	2.1 (2.0, 2.2)	2.1 (2.1, 2.2)	**0.048**
**Duration of continuation phase (months, mean (95% CI))**	5.1 (4.8, 5.5)	4.8 (4.6, 5.0)	4.9 (4.7, 5.0)	0.054
**Treatment facility**				
Government primary health care facility	4 (11.1)	15 (10.2)	19 (10.4)	0.126
Government hospital	31 (86.1)	105 (71.4)	136 (74.3)	
Faith based health center or hospital	0 (0.0)	15 (10.2)	15 (8.2)	
Other	1 (2.8)	12 (8.2)	13 (7.1)	
**Mode of supervision for TB treatment: intensive phase**				
Hospitalized	21 (100.0)	72 (94.7)	93 (95.9)	0.562
Self-administered	0 (0.0)	2 (2.6)	2 (2.1)	
Unknown	0 (0.0)	2 (2.6)	2 (2.1)	
**Mode of supervision for TB treatment: continuation phase**				
Hospitalized	0 (0.0)	2 (2.8)	2 (2.3)	0.609
Self-administered	13 (86.7)	57 (80.3)	70 (81.4)	
Community-based DOT	1 (6.7)	10 (14.1)	11 (12.8)	
Facility-based DOT	1 (6.7)	1 (1.4)	2 (2.3)	
Unknown	0 (0.0)	1 (1.4)	1 (1.2)	
**Currently hospitalized**				
No	15 (41.7)	73 (49.7)	88 (48.1)	0.390
Yes	21 (58.3)	74 (50.3)	95 (51.9)	
**Previously hospitalized in the current phase**				
No	30 (83.3)	117 (79.6)	147 (80.3)	0.613
Yes	6 (16.7)	30 (20.4)	36 (19.7)	
**Number of days hospitalized in the current phase, mean (95% CI)**	41.6 (33.6, 49.5)	43.6 (39.6, 47.7)	43.2 (39.7, 46.8)	0.647

*Abbreviations*: *DOT* Directly observed therapy; *DST* Drug susceptibility testing; *DS TB* Drug susceptible TB; *EPTB* Extra pulmonary TB; *PTB* Pulmonary TB; *TB* Tuberculosis. * *P* values compare the proportions of patients with the characteristic of interest, in the pulmonary and extra-pulmonary groups

### Time lost for care seeking and treatment

Patients lost a mean of 354.4 working hours (95%CI: 287.3, 421.4). Additionally, the household lost a mean of 105.7 working hours (95%CI: 33.1, 178.3) for TB care seeking and treatment (Table 3). Patients with EPTB lost more time compared to PTB patients (490.7 h for EPTB and 321.0 h for PTB, *p* = 0.047) due to a significantly longer time for hospitalization (463.7 h for EPTB versus 292.3 h for PTB, *p* = 0.041).

**Table 3:** Mean number of working hours lost for tuberculosis care for patients and their households in the Solomon Islands national tuberculosis patient cost survey: 2017–2019

Amount of lost time	Extra-pulmonary TB ***N*** = 36 Mean no. of hours (95% CI)	Pulmonary TB ***N*** = 147 Mean no. of hours (95% CI)	All patients ***N*** = 183 Mean no. of hours (95% CI)	***p*** value
**For TB care for TB patients**				
Pre-disease	6.8 (2.0, 11.5)	7.3 (3.7, 11.0)	7.2 (4.2, 10.2)	0.875
Hospitalization	463.7 (295.7, 631.7)	292.3 (221.2, 363.3)	326.0 (260.0, 392.0)	**0.041**
Directly observed therapy	3.4 (−1.6, 8.3)	11.5 (−0.4, 23.5)	9.9 (0.3, 19.6)	0.508
Drug pick-up	15.0 (−2.6, 32.7)	8.2 (6.0, 10.3)	9.5 (5.7, 13.3)	0.152
Follow-up	3.8 (0.8, 6.8)	4.2 (2.0, 6.4)	4.1 (2.2, 6.0)	0.877
**Total lost time**	490.7 (320.6, 660.9)	321.0 (248.6, 393.4)	354.4 (287.3, 421.4)	**0.047**
For TB care for household members of TB patients*				
Hospitalization	103.2 (−5.8, 212.2)	227.3 (13.3, 441.2)	198.5 (33.3, 363.7)	0.531
Directly observed therapy	0.0 (0.0, 0.0)	15.1 (−7.0, 37.3)	12.4 (−5.7, 30.6)	0.531
Drug pick-up	28.5 (−19.2, 76.1)	8.5 (4.6, 12.5)	11.9 (3.9, 19.9)	0.065
Follow-up	1.2 (−0.1, 2.5)	3.8 (1.0, 6.6)	3.2 (1.1, 5.4)	0.312
**Total lost time**	63.4 (9.6, 117.2)	117.6 (25.6, 209.5)	105.7 (33.1, 178.3)	0.544

*Abbreviations*: *CI* Confidence interval; *no* number; *TB* Tuberculosis.* This is in addition to the TB patient

### Estimated total costs incurred by TB patients and their households and proportion of TB-affected households experiencing catastrophic costs (using the output method)

**Fig. 1:**
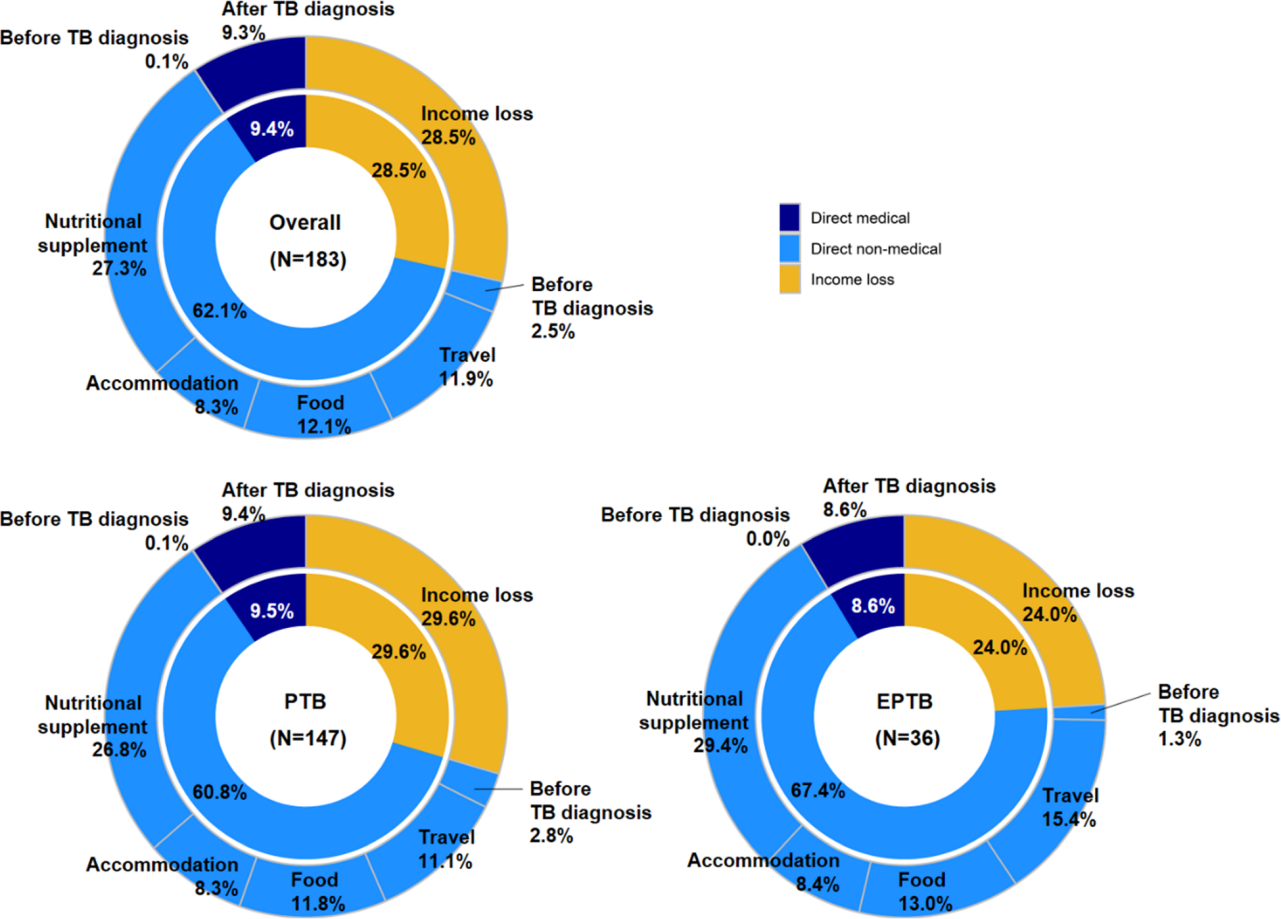
Categories of costs as a proportion of all costs associated with a tuberculosis diagnosis and care from the Solomon Islands national tuberculosis patient cost survey: 2017–2019 (based on mean values)

The median cost of a TB diagnosis and care was USD 716 (IQR: 349–1247), equivalent to 8.8 times the reported monthly household income (Table 4). The costs were driven by direct non-medical costs (62.1%), income loss (28.5%), and direct medical costs (9.4%) (Fig. 1). Costs for nutritional supplements other than the patients’ regular diet accounted for the highest proportion of direct non-medical costs (27.3% of all costs) followed by food costs (12.1%), travel costs (11.9%) and accommodation (8.3%). Direct medical costs were predominantly incurred after diagnosis and were driven by costs associated with hospitalization. There were statistically significant differences observed in the costs of direct medical costs before diagnosis (*p* = 0.033), transport during TB treatment (*p* < 0.001), food during TB treatment (*p* = 0.039), total direct non-medical costs (*p* = 0.044) and direct medical costs (*p* = 0.042) when comparing patients with EPTB and PTB. In all cases, the costs were higher for patients with EPTB, compared to patients with PTB.

**Table 4:** Estimated median costs (USD) incurred by tuberculosis affected households in the Solomon Islands tuberculosis patient cost survey: 2017–2019

Cost category	Extra-pulmonary TB ***N*** = 36 Median (IQR) in USD	Pulmonary TB ***N*** = 147 Median (IQR) in USD	All patients ***N*** = 183 Median (IQR) in USD	***p*** value
**Before TB diagnosis**				
Direct medical	0 (0, 0)	0 (0, 0)	0 (0, 0)	**0.033**
Direct non-medical	6 (5, 10)	6 (4, 6)	6 (4, 6)	0.577
***Total direct before diagnosis***	6 (5, 10)	6 (4, 6)	6 (5, 6)	0.706
**During TB treatment: direct medical**				
Drug pickup	0 (0, 0)	0 (0, 0)	0 (0, 0)	0.776
Directly observed therapy	0 (0, 0)	0 (0, 0)	0 (0, 0)	0.675
Follow up	0 (0, 0)	0 (0, 0)	0 (0, 0)	0.843
Hospitalization	58 (0, 75)	33 (0, 62)	36 (0, 68)	0.181
***Total direct medical during treatment***	58 (0, 76)	36 (0, 69)	37 (0, 71)	0.260
**During TB treatment: direct non-medical**				
Transportation	76 (65, 167)	30 (10, 107)	46 (13, 123)	**< 0.001**
Accommodation	58 (0, 72)	31 (0, 59)	32 (0, 65)	0.109
Food during visits	75 (59, 160)	49 (11, 134)	64 (15, 142)	**0.039**
Nutritional supplement	320 (0, 880)	64 (0, 427)	85 (0, 427)	0.097
***Total direct non-medical***	501 (212, 1323)	395 (122, 826)	435 (146, 961)	**0.046**
**Total**				
Direct medical	58 (0, 76)	36 (0, 70)	38 (0, 73)	0.292
Direct non-medical	509 (218, 1330)	390 (113, 846)	435 (141, 961)	**0.042**
Total direct	630 (271, 1390)	486 (165, 989)	494 (189, 1110)	**0.044**
Income loss	99 (0, 258)	74 (0, 333)	74 (0, 296)	0.500
***Grand total***	779 (427, 2302)	693 (343, 1207)	716 (349, 1247)	0.107

*Abbreviations*: *IQR* Inter quartile range; *EPTB* Extra pulmonary TB; *PTB* Pulmonary TB; *USD* United States Dollars; *TB* Tuberculosis

The proportion of TB-affected households experiencing catastrophic costs was 92.3% (95%CI: 88.5–96.2%) using the output approach. When only direct costs were included (which accounted for 71.5% of total TB patient costs overall), 81.4% of TB-affected households incurred catastrophic costs. When the catastrophic costs threshold was changed (in a range from 10 to 60%, using the output approach), the proportion of patients incurring catastrophic costs ranged from 78.1 to 95.1% (Supplementary Table 3).

### Influence of adjustment for cash-based household income on estimated total costs incurred by TB patients and their households and proportion of TB-affected households experiencing catastrophic costs

We also estimated income and therefore the proportion of patients with catastrophic costs taking into account the reported household income observed in this survey and the household income decile recorded in the Solomon Islands HIES. Using this approach 171 (93.4%) patients were classified in the poorest wealth decile. The remainder were categorized as being in the second to fourth wealth decile groups, with no households classified in the fifth to tenth deciles (Supplementary Table 4). The adjusted mean monthly household income was then estimated at USD 367.9 (95%CI: 298.6–437.3) and the median cost of TB care was USD 1193 (IQR: 532–2346), with a corresponding increase in income loss to USD 312 (IQR: 0–1247). This was equivalent to 3.2 times the adjusted monthly household income and 14.6 times the reported median monthly household income (based on the survey responses). Costs were higher among patients with EPTB (USD 1202; IQR: 727–2777) than patients with PTB (USD 1107; IQR: 521–2241, *p* = 0.195) (Supplementary Table 5). Using the adjusted household income, the proportion of direct non-medical costs was 48.1% (versus 62.1% using the output method), while the proportion of income loss increased from 28.5 to 45.4%, and the proportion of direct medical costs was 6.4%, compared 9.4% using the output method. Then, the proportion of TB-affected households facing catastrophic costs was 82.0% (95%CI: 76.3–87.6%; 86.1% (95%CI: 74.2–98.0%) for patients with EPTB, and 81.0% (95%CI: 74.5–87.4%) for patients with PTB).

### Factors associated with catastrophic costs due to TB

In multivariate analyses, where we used the adjusted household income, being poorer was significantly associated with having catastrophic costs due to TB (Table 5). 96.9% of TB affected households classified as being in the first (poorest), second or third poorest quintiles faced catastrophic costs, and they had 67.3 times higher odds of experiencing catastrophic costs compared with the wealthiest households (95% CI: 15.86–489.74, *p* < 0.001).

**Table 5:** Risk factors associated with catastrophic costs due to tuberculosis from the Solomon Islands national tuberculosis patient cost survey: 2017–2019

Variable and categories		N	n (%)	Crude OR (95% CI, *p*-value)	Adjusted OR (95% CI, *p*-value)
Age group (years)	0–14	21	16 (76.2)	Referent	–
	15–24	41	34 (82.9)	1.52 (0.40–5.52, *p* = 0.527)	1.80 (0.21–15.57, *p* = 0.587)
	25–34	40	30 (75.0)	0.94 (0.26–3.14, *p* = 0.918)	1.04 (0.15–6.89, *p* = 0.966)
	35–44	38	31 (81.6)	1.38 (0.36–5.05, *p* = 0.623)	1.04 (0.15–6.83, *p* = 0.964)
	45–54	24	22 (91.7)	3.44 (0.65–26.19, *p* = 0.170)	1.59 (0.16–19.20, *p* = 0.694)
	55+	19	17 (89.5)	2.66 (0.49–20.44, *p* = 0.281)	0.79 (0.08–8.97, *p* = 0.839)
Sex	Female	91	73 (80.2)	Referent	–
	Male	92	77 (83.7)	1.27 (0.59–2.73, *p* = 0.541)	2.81 (0.91–9.59, *p* = 0.082)
Type of disease	EPTB	36	31 (86.1)	Referent	–
	PTB	147	119 (81.0)	0.69 (0.22–1.79, *p* = 0.473)	0.41 (0.08–1.69, *p* = 0.241)
Income quintile	Wealthiest	48	21 (43.8)	Referent	–
	Fourth	38	35 (92.1)	15.00 (4.60–68.37, *p* < 0.001)	17.66 (4.81–94.42, *p* < 0.001)
	Poorest, second or third	97	94 (96.9)	40.29 (12.77–180.20, *p* < 0.001)	67.25 (15.86–489.74, *p* < 0.001)
Household size (no. of persons)	0–7	91	75 (82.4)	Referent	–
	8 or more	92	75 (81.5)	0.94 (0.44–2.01, *p* = 0.875)	–
Education level	University, vocational or other	10	6 (60.0)	Referent	–
	Secondary school	49	35 (71.4)	1.67 (0.38–6.77, *p* = 0.477)	2.37 (0.30–19.05, *p* = 0.408)
	No education, pre-school, or primary school	123	109 (88.6)	5.19 (1.21–20.55, *p* = 0.020)	5.18 (0.69–38.16, *p* = 0.102)
Type of health facility	Government primary health care facility	19	17 (89.5)	Referent	–
	Government hospital	136	108 (79.4)	0.45 (0.07–1.71, *p* = 0.309)	–
	Faith based health center or hospital	15	13 (86.7)	0.76 (0.08–7.07, *p* = 0.801)	–
	Other	13	12 (92.3)	1.41 (0.12–32.44, *p* = 0.788)	–
TB treatment phase	Intensive phase	97	83 (85.6)	Referent	–
	Continuation phase	86	67 (77.9)	0.59 (0.27–1.27, *p* = 0.181)	0.27 (0.02–4.05, *p* = 0.319)
Hospitalized in current phase	No	147	122 (83.0)	Referent	–
	Yes	36	28 (77.8)	0.72 (0.30–1.85, *p* = 0.467)	–
Hospitalized at time of interview	No	88	68 (77.3)	Referent	–
	Yes	95	82 (86.3)	1.86 (0.87–4.08, *p* = 0.115)	0.70 (0.05–10.76, *p* = 0.783)
Delay before diagnosis	No	39	35 (89.7)	Referent	–
	Yes	44	37 (84.1)	0.60 (0.15–2.18, *p* = 0.452)	–

*Abbreviations*: *CI* Confidence interval; *OR* Odds ratio

### Reported coping mechanism and social consequences

Overall, 19.7% (*n* = 36) of TB-affected households used their savings, borrowed money, or sold their assets as financial coping mechanisms (Table 6). One quarter (24.6%, *n* = 45) experienced food insecurity, 21.3% (*n* = 39) interrupted schooling, and 8.2% lost their job (*n* = 15). In terms of perceived financial impact due to TB, 83.7% (*n* = 153) reported that TB had a moderate to very serious financial impact on their households (moderate: 37.2%, serious: 35.0%, very serious: 11.5%; these categories of severity were self reported by patients). Only 4.9% (*n* = 9) received social support either from family or relatives (*n* = 7) or from the Parliament (*n* = 2). In addition, 36.1% (*n* = 66) received vouchers in the form of food support (*n* = 63), a travel voucher (*n* = 1), or via an unknown source (*n* = 2). There were no statistically significant differences observed between patients with EPTB or PTB for coping mechanisms, social consequences, social support or receipt of vouchers.

**Table 6:** Coping mechanisms, social consequences, perceived financial impact and social support reported by patients who participated in the Solomon Islands tuberculosis patient cost survey: 2017–2019

Category	Extra-pulmonary TB ***N*** = 36 (%) n (%)	Pulmonary TB ***N*** = 147 n (%)	All patients ***N*** = 183 n (%)	***p*** value
**Coping mechanisms**				
Dissavings	2 (5.6)	15 (10.2)	17 (9.3)	0.389
Took out a loan	3 (8.3)	17 (11.6)	20 (10.9)	0.578
Sold assets	0 (0.0)	6 (4.1)	6 (3.3)	0.218
Any of the above	5 (13.9)	31 (21.1)	36 (19.7)	0.330
**Social consequences**				
Social exclusion	2 (5.6)	12 (8.2)	14 (7.7)	0.598
Food insecurity	7 (19.4)	38 (25.9)	45 (24.6)	0.424
Job loss	5 (13.9)	10 (6.8)	15 (8.2)	0.165
Interrupted schooling	7 (19.4)	32 (21.8)	39 (21.3)	0.760
Divorce	0 (0.0)	1 (0.7)	1 (0.5)	0.620
Any of the above	20 (55.6)	79 (53.7)	99 (54.1)	0.845
**Perceived financial impact**				
No impact	1 (2.8)	0 (0.0)	1 (0.5)	0.205
Little impact	3 (8.3)	26 (17.7)	29 (15.8)	
Moderate impact	14 (38.9)	54 (36.7)	68 (37.2)	
Serious impact	14 (38.9)	50 (34.0)	64 (35.0)	
Very serious impact	4 (11.1)	17 (11.6)	21 (11.5)	
**Social support**				
From family or relatives	1 (50.0)	6 (85.7)	7 (77.8)	0.284
From a Member of Parliament	1 (50.0)	1 (14.3)	2(22.2)	
***Total (proportion of all patients)***	2 (5.6%)	7 (4.8)	9 (4.9)	0.844
**Receipt of vouchers**				
Food support	16 (100.0)	47 (94.0)	63 (95.5)	0.605
Travel voucher	0 (0)	1 (2.0)	1 (1.5)	
Unknown type	0 (0)	2 (4.0)	2 (3.0)	
***Total (proportion of all patients)***	16 (44.4)	50 (34.0)	66 (36.1)	0.243

*Abbreviation*: *TB* Tuberculosis

## Discussion

This survey identified that the cost of TB care is catastrophic for almost all TB-affected households in Solomon Islands (92.3%, or 82.0%, adjusted) as the median cost of TB care was equivalent to 8.8 times the monthly household income. Only 5 % of patients received social support during their TB care, only 2 % of patients had health insurance and one in five used their savings, borrowed money, or sold their assets to cope with the economic burden of TB.

The global TB report published by WHO in 2020 provides an overview of the results of other TB patient cost surveys to compare to the results observed in Solomon Islands [19]. By July 2019, 17 countries had completed a national TB patient cost survey [19]. In these countries, the percentage of TB-affected households who experienced catastrophic costs ranged from 19% (95%CI: 15–25%) in Lesotho to 83% (95%CI: 76–86) in Timor-Leste [19, 20]. Overall, the pooled average of catastrophic costs was 49% (95%CI: 34–63) [19]. The figure observed in Solomon Islands is higher than the figures reported in all of these countries.

The high proportion of direct non-medical costs in Solomon Islands (62.1% of all costs) was largely driven by nutritional supplements (corresponding to the largest group of non-medical costs, at 27.3% of all costs). A similar observation was made in the Timor-Leste national TB patient cost survey, and in other surveys [20–22]. Malnutrition is a known risk factor for TB [10] and weight loss is a well-documented symptom of TB [23]. Therefore there may be some groups of patients who are malnourished or underweight at TB diagnosis who seek to use nutritional supplements to recover. However, it is not clear what nutritional supplements are being bought by TB patients, if they have been recommended by health care workers, or if there are other local beliefs about the effect of certain nutritional supplements on health. The proportion of patients who are malnourished or who are underweight at TB diagnosis is also unknown in the Solomon Islands context. These issues require further investigation.

Travel, food and accommodation costs were also considerable, accounting for 32.3% of all costs, which may be attributable to the fact that the people travel (often long distances) to get to one of the DOTS centres for a TB diagnosis. TB care is offered in one DOTS centre per province and the country is very geographically dispersed. Therefore patients and their families may travel long distances from their home village to the nearest DOTS centre to seek a TB diagnosis. If they are diagnosed, then the current national policy is that they are hospitalised for the first two months of TB care (i.e. the intensive phase). This is reflected in our results where approximately half (51.9%) of patients were hospitalised at the time of interview and one in five patients (19.7%) had been previously hospitalised. The Ministry of Health and Medical Services is currently considering options to introduce community based and ambulatory treatment.

Income loss accounted for 28.5% of all costs and 8.2% of people lost their job while receiving TB care. Loss of employment and income loss are major barriers faced by many TB patients, [22, 24, 25] with the stigma around TB and misunderstandings about infectiousness as potential contributing factors. These income losses can be compensated for by social or financial protection schemes in many countries. However, these kinds of schemes are generally not available in the Solomon Islands. Voluntary health insurance is also generally not available with the exception of schemes for expatriates [15].

### Strengths and limitations

This is the first survey on the costs associated with TB diagnosis and care in Solomon Islands. The survey provided baseline information including information on health seeking behaviour, the costs of health care and the drivers of these costs; these data can be used for further monitoring of the *End TB Strategy* indicator and for national planning. The survey investigators also demonstrated that collecting data using online tools is feasible in the Solomon Islands context and data completeness was attained for many variables. In addition, the survey is nationally representative, including all ten DOTS centres, which is not always easy in geographically dispersed countries such as the Solomon Islands.

This survey also has some limitations. First, the survey investigators originally intended to recruit patients from April to November 2016 and had developed a sampling strategy that included a representative sample from all provinces in the Solomon Islands, whereas, in practice, the period of recruitment ran over three years (2017–2019). However, a comparison of some of the key demographic characteristics in the sample was undertaken and these were compared to the 2018 national TB surveillance data. There were no statistically significant differences in these variables when comparing the two populations (T. Yamanaka, personal communication, 2020). Second, this was a cross-sectional survey with forward extrapolations on patient costs. While this is a practical method given the context of the study and is recommended by WHO, the estimated costs may not reflect the true costs experiences by the patients due to the illness. Longitudinal methods of determining patient costs are accurate but may be more accurate. Third, recall bias is a concern when recalling costs incurred in the past. As much as possible the interviewers used prompts to assist patients in recalling costs, but it is possible that the recollected numbers may have been inaccurate. Fourth, the survey did not reach the estimated sample size, but it was planned on notifications in previous years and then there were relatively small numbers of patients in the participating DOTS centres, a common challenge in countries with a lower TB case load and where the number of TB patients may fluctuate over time. As well, we recalculated the sample size based on our finding that the proportion of patients with catastrophic costs in Solomon Islands was 92.3%. Using this figure and the other sample size parameters described in our Methods section, we would have required a sample size of 126 TB patients. Therefore our sample size of 183 TB patients was sufficient even though it fell short of our original estimated sample size. Then, we used an estimate of 30% of catastrophic costs to estimate our sample size, which was an under estimate. However using a higher estimate would have resulted in a smaller sample size, so we did not under sample in our study and as more catastrophic cost surveys have been conducted now it may be more feasible to provide an estimate of the patients with catastrophic costs. Lastly, self-reported income was used as the measure of income in this survey. Other methods of reporting or estimating income - such as household consumption/ expenditure - may be more accurate in settings such as Solomon Islands where the majority of the population are employed in the informal employment sector. Therefore, the proportion of patients with catastrophic costs may have been overestimated in this survey, which is consistent with the fact that only 11.5% of people thought that TB had a very severe financial impact on them. Future methodological work on measuring income may determine which approach is preferred [26].

### Public health implications

The findings from this survey highlight some of the difficulties that patients in Solomon Islands experience when accessing TB care. These findings are informative for policy makers as they consider how they can improve and expand universal health coverage in the Solomon Islands. One obvious policy relevant finding is the cost of accessing TB care in Solomon Islands and the need to mitigate these costs of care, where possible. Mitigation strategies may include the provision of vouchers or subsidies for TB patients that could be provided by the NTLP in the absence of wider social or financial protection schemes, which are currently limited in the Solomon Islands [27]. Recently, the Solomon Islands Government has adopted universal health coverage as the principle of health system design and a role delineation policy is the major vehicle to achieve it [11]. This policy defines a service delivery package and ensures minimum services at the lowest level of health services close to the community [11]. In the years to come, universal health coverage rollout in Solomon Islands may help to mitigate patient costs. A small financial incentive has been part of the Global Fund TB grant in the Solomon Islands for a number of years. However, there have been problems with operationalising this scheme and it may now be timely to revisit the reasons for this and propose a workable solution. The cost of TB care also has an impact on families and given the system of family responsibility in Solomon Islands as part of the wantok system, [28] a kinship network where caring for one’s relatives is extremely important, the impact of TB on families (especially poor ones) should be recognised. In addition, over the longer term any social or financial protection schemes should be inclusive of TB patients and future models of TB care should recognise the costs of care as a factor in their planning.

## Conclusion

Tuberculosis is a financially disabling condition in Solomon Islands. Non-medical costs and loss of income are the main cost drivers. TB patients had very little access to social or financial protection and not being in the wealthiest wealth quintile was associated with catastrophic costs. In the longer term access to social and financial protection may protect patients against the costs of TB care. However, in the shorter term the NTLP may want to consider the provision of vouchers to TB patients and their families while longer term measures such as the advancement of universal health coverage and other social and financial protection measures are pursued.

## Supplementary Information

**Additional file 1.** National TB patient cost survey questionnaire for the Solomon Islands.

**Additional file 2: Table S1.** Household income by type of income and by decile in the Solomon Islands Household Income and Expenditure Survey, 2013 (in Solomon Islands dollars). **Table S2.** Mean number of facility visits and diagnostic delay for participants in the Solomon Islands national tuberculosis patient cost survey: 2017–2019. **Table S3.** Tuberculosis affected households classified as facing catastrophic costs by different thresholds, from the Solomon Islands national tuberculosis patient cost survey: 2017–2019. **Table S4.** Re-classification of tuberculosis-affected households into income decile based on the 2013 Solomon Islands Household Income and Expenditure Survey. **Table S5.** Estimated median total costs (USD) incurred with adjusted household income from the Solomon Islands national tuberculosis patient cost survey: 2017–2019.

## Data Availability

The datasets used and/or analysed during the current study are available from the corresponding author on reasonable request, and following approval by the Solomon Islands Ministry of Health and Medical Services, who are the custodians of the data. The questionnaire was developed by the Global Tuberculosis Programme, World Health Organization and we have permission to publish it.

## Abbreviations

DOTS: Directly Observed Teatment Short-course
EPTB: Extra pulmonary tuberculosis
HIES: Household Income and Expenditure Survey
IQR: Inter quartile range
NTLP: National tuberculosis and leprosy programme
PTB: Pulmonary tuberculosis
TB: Tuberculosis
USD: United States dollars
WHO: World Health Organization
