# Correction to: Economic evaluation of patient costs associated with tuberculosis diagnosis and care in Solomon Islands

**DOI:** 10.1186/s12889-021-12284-5

**Published:** 2021-12-30

**Authors:** Kerri Viney, Noel Itogo, Takuya Yamanaka, Ridha Jebeniani, Anupama Hazarika, Fukushi Morishita, Nobuyuki Nishikiori, Susana Vaz Nery

**Affiliations:** 1 Research School of Population Health, Australian National University, Canberra, Australia; 2 Global TB Programme, World Health Organization Headquarters, Geneva, Switzerland; 3 Department of Global Public Health, Karolinska Institutet, Stockholm, Sweden; 4 National TB Programme, Ministry of Health and Medical Services, Honiara, Solomon Islands; 5 World Health Organization Country Office, Honiara, Solomon Islands; 6 Department of Global Health and Development, London School of Hygiene & Tropical Medicine, London, UK; 7 School of Tropical Medicine & Global Health, Nagasaki University, Nagasaki, Japan; 8 Independent consultant, Tunis, Tunisia; 9 End TB and Leprosy Unit, World Health Organization Regional Office of the Western Pacific, Manila, Philippines; 10 The Kirby Institute, University of New South Wales Sydney, Sydney, Australia

**Correction to: BMC Public Health 21, 1928 (2021)**

**https://doi.org/10.1186/s12889-021-11938-8**

It was highlighted that in the original article [1] the Authors’ Contributions section was incomplete. This Correction article shows the correct Authors’ Contributions section. The original article has been updated.

**Authors’ contributions**

KV and SVN submitted an initial funding application for the study. KV, SVN, NI and NN conceived the study. NI, KV, SVN, RJ and NN designed the study. NI, KV, SVN, RJ, FM, AH and TY were involved in the implementation of the study, which was led by NI and the Solomon Islands NTLP. TY analysed the data. All authors interpreted the data. KV drafted the manuscript. All authors read, critically revised and approved the final manuscript. The authors alone are responsible for the views expressed in this article and they do not necessarily represent the views, decisions or policies of the institutions with which they are affiliated.
