# Supplementary material for: Economic evaluation of patient costs associated with tuberculosis diagnosis and care in Solomon Islands
Source: BMC Public Health. 2021 Oct 23;21:1928. doi: 10.1186/s12889-021-11938-8 (PMC8542301; doi:10.1186/s12889-021-11938-8)
Supplement: Supplementary file 1 — Additional file 1. National TB patient cost survey questionnaire for the Solomon Islands. [file 12889_2021_11938_MOESM1_ESM.pdf]

Nationwide TB-related catastrophic costs survey in Solomon Islands

Part I A. Patient information to be obtained from TB treatment card before interview

YEAR OF PATIENT REGISTRATION IN TB REGISTER *

- ◯ 2017
- ◯ 2018
- ◯ 2019

PATIENT NUMBER IN TB REGISTER *

ENTER THE WORD TB HERE (IN CAPS) TO CAPTURE THE DIAGNOSIS *

HEALTH FACILITY NAME

- ◯ Taro Hospital
- ◯ Gizo Hospital
- ◯ Helena Goldie Hospital
- ◯ Buala Hospital
- ◯ National Referral Hospital
- ◯ Good Samaritan Hospital
- ◯ Kilu'ufi Hospital
- ◯ Atoifi Hospital
- ◯ Kirakira Hospital
- ◯ Lata Hospital

DATE OF INTERVIEW

yyyy-mm-dd

NAME OF PROVINCE *

- Central
- Choiseul
- Guadalcanal
- Honiara City
- Malaita
- Makira
- Renbel
- Temotu
- Western
- Ysabel

NAME OF WARD

PATIENT ID NUMBER IS

INTERVIEWER NAME

CATEGORY OF TREATING FACILITY (SEVERAL ANSWERS ARE POSSIBLE)

- Government primary health care facility
- Government hospital
- Faith based health center or hospital
- Private clinic or hospital
- Other

SPECIFY OTHER.

ON MDR-TB TREATMENT? *

- Yes
- No

TREATMENT REGISTRATION GROUP *

- 1st line, new
- 1st line, relapse
- 1st line, retreatment after loss to follow-up
- 1st line, retreatment after failure
- Other

SPECIFY OTHER.

TREATMENT REGISTRATION GROUP *

- MDR, new (initial MDR)
- MDR, relapse (after intial or retreatment regimens)
- MDR, re-treatment after loss to follow-up (after intial or retreatment regimens)
- MDR, re-treatment after failure (IR or RR)
- Other

SPECIFY OTHER.

Part II. Informed Consent

AT THIS POINT THE INTERVIEWER SHOULD PROVIDE THE PATIENT WITH THE INFORMATION SHEET, SHOULD EXPLAIN THE STUDY AND SHOULD OBTAIN WRITTEN INFORMED CONSENT

HAS WRITTEN INFORMED CONSENT BEEN OBTAINED?

- Yes
- No

DECISION ABOUT INCLUSION OR EXCLUSION

- Included
- Excluded

IF EXCLUDED, REASON FOR EXCLUSION

- No informed consent
- Treatment registration group is "other" (i.e. answer 5 or 10 in the question: Treatment registration group)
- Unable to get accurate data
- Patient within first two weeks of current treatment phase

INTERVIEWEE IDENTITY

- Patient
- Guardian or Parent (for children aged 0-17 years)
- Other

SPECIFY OTHER.

Part I B. Patient information to be obtained from TB treatment card before interview

SEX
- ◯ Male
- ◯ Female

AGE (IN YEARS)

DATE OF FIRST BACTERIOLOGICAL TB INVESTIGATION

yyyy-mm-dd

DIAGNOSTIC TB TEST USED
- ☐ Smear microsocopy
- ☐ Culture
- ☐ Molecular test (such as Xpert MTB/RIF)
- ☐ Chest x-ray
- ☐ Other

SPECIFY OTHER.

DATE OF DIAGNOSIS

yyyy-mm-dd

PLACE OF DIAGNOSIS
- ◯ Government primary health care facility
- ◯ Government hospital
- ◯ Faith based health center or hospital
- ◯ Private clinic or hospital
- ◯ Other

SPECIFY OTHER.

TYPE OF TB
- ◯ Pulmonary, bacteriologically confirmed
- ◯ Pulmonary, clinically diagnosed
- ◯ Extra-pulmonary, bacteriologically confirmed
- ◯ Extra-pulmonary, clinically diagnosed

DRUG-SUSCEPTIBILITY TEST DONE?
- ◯ Yes
- ◯ No
- ◯ Unknown

IF YES, WHAT WAS THE LATEST TEST?
- ◯ Gene Xpert MTB/Rif
- ◯ LPA
- ◯ Culture with DST
- ◯ Other

SPECIFY OTHER.

IF YES, DRUG SUSCEPTIBLE RESULTS?

- Rif-resistant
- MDR-TB
- Non Rif-resistant or MDR-TB, DR-TB
- Non Rif-resistant or MDR-TB, DS-TB
- Unknown

TREATMENT REGIMEN PRESCRIBED

- Initial regimen (2HRZE/4HR)
- Retreatment regimen (2HRZES/1HRZE/5HRE)
- Childhood regimen (2HRZ/4HR)
- Second-line standardized regimen
- Second-line indiviudalized regimen
- Other

SPECIFY OTHER.

TOTAL DURATION OF PLANNED TREATMENT FROM START (MONTHS)

START DATE OF CURRENT TB TREATMENT

yyyy-mm-dd

THE PATIENT IS CURRENTLY IN THE INTENSIVE OR CONTINUATION TREATMENT PHASE? *

- Intensive phase
- Continuation phase

HOW MANY DAYS OF THIS PHASE HAS THE PATIENT COMPLETED?

HIV STATUS (ONLY IF INDICATED ON CARD)

- HIV positive
- HIV negative
- Not recorded
- Declined
- Unknown

IS THE PATIENT HOSPITALIZED AT THE TIME OF INTERVIEW?

- Yes
- No

IF HOSPITALIZED AT THE TIME OF INTERVIEW, WHEN IS THE PLANNED DATE FOR DISCHARGE?

yyyy-mm-dd

PATIENT SHOULD BE EXCLUDED FROM SURVEY UNTIL NEXT VISIT AS HE/SHE IS IN FIRST TWO WEEKS OF TREATMENT PHASE!

Part III. Overview of TB treatments before current treatment (for re-treatment cases only)

IN THE LAST TWO YEARS, HOW MANY TIMES HAVE YOU BEEN TREATED FOR TB BEFORE THE CURRENT TREATMENT, INCLUDING COMPLETED AS WELL AS NON-COMPLETED TREATMENTS? (ENSURE THAT INFORMATION IS FILLED IN FOR EACH EPISODE OF TB IF THERE IS MORE THAN ONE PREVIOUS EPISODE)

Part IV - Costs before the current TB treatment (for new cases in intensive phase only)

FOR THIS EPISODE OF TB, HOW MANY WEEKS BEFORE TREATMENT STARTED DID YOU FIRST EXPERIENCE SYMPTOMS OF TB?

DID YOU SEEK TREATMENT OR ADVICE FOR SYMPTOMS OF THE CURRENT ILLNESS BEFORE YOU WERE DIAGNOSED WITH TB?

- ◯ Yes
- ◯ No

IF SO, HOW MANY TIMES DID YOU SEEK TREATMENT OR ADVICE FOR YOUR SYMPTOMS BEFORE YOU WERE DIAGNOSED?

WHAT TYPES OF HEALTH CARE FACILITIES DID YOU VISIT WHEN YOU SOUGHT TREATMENT OR ADVICE FOR SYMPTOMS OF THE CURRENT ILLNESS?

- [ ] Government health care facility
- [ ] Government hospital
- [ ] Faith based health center or hospital
- [ ] Private clinic or hospital
- [ ] Pharmacy
- [ ] Kastom doctor
- [ ] Village health worker
- [ ] Other

SPECIFY OTHER.

FIRST HEALTH CARE FACILITY VISIT

1

TYPE OF PROVIDER

- ○ Government health care facility
- ○ Government hospital
- ○ Faith based health center or hospital
- ○ Private clinic or hospital
- ○ Pharmacy
- ○ Kastom doctor
- ○ Village health worker
- ○ Other

SPECIFY OTHER.

TRAVEL TIME FOR VISIT (IN HOURS)	TIME SPENT FOR VISIT (IN HOURS)	DAY CHARGES (FOR HOSPITALIZATIONS ONLY)	CONSULTATION FEE
RADIOGRAPHY AND OTHER IMAGING FEES	LAB TEST FEES	OTHER PROCEDURES	MEDICINE FEES

OTHER INCLUDING NUTRITIONAL SUPPLEMENTS	TOTAL MEDICAL COSTS (IF CANNOT DISAGGREGATE)	TRAVEL (RETURN) COSTS	FOOD COSTS

OTHER NON-MEDICAL (INCLUDING ACCOMODATION)	TOTAL NON-MEDICAL COSTS (IF CANNOT DISAGGREGATE)	INSURANCE REIMBURSEMENT

CLICK "+" TO ADD ADDITIONAL VISIT

Part V. Cost during current TB/MDR-TB treatment (to be filled for all patients)

ARE YOU CURRENTLY HOSPITALIZED?

- ◯ Yes
- ◯ No

HAVE YOU BEEN PREVIOUSLY HOSPITALIZED DURING YOUR CURRENT TB TREATMENT PHASE AND BECAUSE OF TB?

- ◯ Yes
- ◯ No

HOW MANY TIMES?

FIRST HOSPITAL STAY DURING CURRENT TREATMENT PHASE BECAUSE OF TB 1

TYPE OF HOSPITAL

- ◯ Government primary health care facility
- ◯ Government hospital
- ◯ Faith based health center or hospital
- ◯ Private clinic or hospital
- ◯ Other

SPECIFY OTHER.

NUMBER OF DAYS HOSPITALIZED?

TRAVEL TIME TO HOSPITAL (IN HOURS)?

HOW MUCH IN TOTAL DID YOU SPEND ON THE FOLLOWING CATEGORIES IN THIS HOSPITAL STAY:

DAY CHARGES

CONSULTATIONS

RADIOGRAPHY AND OTHER IMAGING

LAB TESTS

PROCEDURES (INCLUDING SURGERY, BIOPSY)

MEDICINES FEES

OTHER, INCLUDING NUTRITIONAL SUPPLEMENT

TOTAL MEDICAL (IF CANNOT DISAGGREGATE)

TRAVEL (RETURN) COSTS

FOOD COSTS

OTHER NON-MEDICAL (INCLUDING ACCOMMODATION)

TOTAL NON-MEDICAL (IF CANNOT DISAGGREGATE)

TOTAL OUT-OF-POCKET PAYMENTS PER STAY (IF CANNOT DISAGGREGATE)

HEALTH INSURANCE REIMBURSEMENT

CLICK "+" TO ADD ADDITIONAL STAY

COSTS FOR DOT AND FOOD COSTS DURING AMBULATORY CARE

ON A DAILY BASIS, DO YOU CURRENTLY TAKE YOUR MEDICINES YOURSELF WITHOUT SUPERVISION OR SUPPORT (SELF-ADMINISTERED) OR DO YOU HAVE A TREATMENT SUPERVISOR OR SUPPORTER (DOT)?

- Self-administered
- Facility based DOT
- Community based DOT

WHO IS THE DOT PROVIDER/SUPPORTER?

- Government health facility
- Private health facility
- Community volunteer
- Other

SPECIFY OTHER.

HOW LONG DID THE LAST DOT VISIT TAKE, INCLUDING TRAVEL TIME AND WAITING TIME (TOTAL TURNAROUND TIME) IN HOURS?

WHAT WAS THE COST OF TRANSPORT (RETURN) FOR THE LAST DOT VISIT, IN TOTAL FOR YOU AND ANY ACCOMPANYING HOUSEHOLD MEMBER?

HOW MUCH DID YOU SPEND ON FOOD AND DRINKS FOR THE LAST DOT VISIT (ON THE ROAD, WHILE WAITING, LUNCH ETC.), IN TOTAL FOR YOU AND ANY ACCOMPANYING HOUSEHOLD MEMBER?

HOW MUCH (IF ANY) WAS PAID TO DOT PROVIDER BY THE PATIENT AT THE LAST DOT VISIT (INCLUDING IN-KIND)?

COSTS OF PICKING UP DRUGS AND FOOD COSTS DURING AMBULATORY CARE

DO YOU OR A HOUSEHOLD MEMBER PICK UP TB DRUGS (FOR SELF-ADMINISTERED TREATMENT OR TO BRING TO YOUR DOT SUPERVISOR/SUPPORTER)?

- Yes
- No

HOW OFTEN DO YOU OR A HOUSEHOLD MEMBER PICK UP TB DRUGS IN THE CURRENT TREATMENT PHASE?

- Every week
- Every two weeks
- Every month
- Other

SPECIFY OTHER.

WAS THERE A FEE PAID (INCLUDING IN KIND) TO PICK UP DRUGS? (INLUDING FEES PAID BY PATIENT TO COMMUNITY VOLUNTEER OR OTHERS TRAVELLING TO THE FACILITY TO PICKUP DRUGS)

- Yes
- No

HOW MUCH?

WHERE DO YOU OR YOUR HOUSEHOLD MEMBER PICK UP YOUR TB DRUGS?

- Government health care facility
- Government hospital
- Faith based health center or hospital
- Private clinic or hospital
- Pharmacy
- Kastom doctor
- Village health worker
- Other

SPECIFY OTHER.

WHAT ACCOMMODATION COST DID YOU AND ANY ACCOMPANYING HOUSEHOLD MEMBER HAVE WHEN YOU LAST PICKED UP DRUGS?

HOW LONG DID THE LAST VISIT TO PICK UP DRUGS TAKE, INCLUDING TRAVEL TIME AND WAITING TIME (TOTAL TURNAROUND TIME) IN HOURS?

WHAT WAS THE COST OF TRANSPORT (RETURN) LAST TIME YOU PICKED UP DRUGS, INCLUDING PARKING COSTS, IN TOTAL FOR YOU AND ANY ACCOMPANYING HOUSEHOLD MEMBER?

HOW MUCH DID YOU SPEND ON FOOD AND DRINKS LAST TIME YOU PICKED UP DRUGS (ON THE ROAD, WHILE WAITING, LUNCH ETC.), IN TOTAL FOR YOU AND ANY ACCOMPANYING HOUSEHOLD MEMBER?

COST DURING OUTPATIENT VISITS FOR MEDICAL FOLLOW-UP (E.G. TO SEE THE DOCTOR OR NURSE, OR TO HAVE TESTS)

HOW MANY TB-RELATED MEDICAL FOLLOW-UP VISITS HAVE YOU HAD SO FAR DURING THIS TREATMENT PHASE (TO SEE THE DOCTOR OR NURSE, HAVE FOLLOW- UP TESTS, ETC.)?

HOW LONG DID THE LAST FOLLOW-UP MEDICAL OUTPATIENT VISIT TAKE, INCLUDING TRAVEL TIME AND WAITING TIME (TOTAL TURNAROUND TIME) IN HOURS?

WHAT WAS THE COST OF TRANSPORT (RETURN) AT THE LAST FOLLOW-UP MEDICAL OUTPATIENT VISIT IN TOTAL FOR YOU AND ANY ACCOMPANYING HOUSEHOLD MEMBER?

WHAT ACCOMMODATION COST DID YOU HAVE FOR THE LAST VISIT, IN TOTAL, FOR YOU AND ANY ACCOMPANYING HOUSEHOLDMEMBER?

HOW MUCH DID YOU PAY DURING YOUR LAST FOLLOW-UP MEDICAL OUTPATIENT VISIT FOR REGISTRATION/CONSULTATION?

HOW MUCH DID YOU PAY DURING YOUR LAST FOLLOW-UP MEDICAL OUTPATIENT VISIT FOR RADIOGRAPHY AND OTHER IMAGING?

HOW MUCH DID YOU PAY DURING YOUR LAST FOLLOW-UP MEDICAL OUTPATIENT VISIT FOR TESTS, TB TESTS AND OTHERS?

HOW MUCH DID YOU PAY DURING YOUR LAST FOLLOW-UP MEDICAL OUTPATIENT VISIT FOR OTHER PROCEDURES?

HOW MUCH DID YOU PAY AT YOUR LAST FOLLOW-UP MEDICAL OUTPATIENT VISIT FOR TB MEDICINES, INCLUDING PRESCRIPTIONS FOR MEDICINES BOUGHT OUTSIDE THE FACILITY?

HOW MUCH DID YOU PAY DURING YOUR LAST FOLLOW-UP MEDICAL OUTPATIENT VISIT FOR OTHER MEDICINES, INCLUDING NUTRITIONAL SUPPLEMENTS?

WHAT OTHER FEES NOT LISTED IN THE PREVIOUS QUESTIONS DID YOU PAY DURING YOUR LAST FOLLOW-UP MEDICAL OUTPATIENT VISIT (INCLUDING DONATION)?

WHAT ARE SOME LOCAL EXAMPLES OF OTHER FEES THAT YOU ENCOUNTERED DURING YOUR LAST FOLLOW-UP MEDICAL OUTPATIENT VISIT?

COSTS FOR NUTRITIONAL/FOOD SUPPLEMENTS

DO YOU BUY ANY NUTRITIONAL SUPPLEMENTS OUTSIDE YOUR REGULAR DIET BECAUSE OF THE TB ILLNESS, FOR EXAMPLE VITAMINS, MEAT, ENERGY DRINKS, OR FRUITS AS RECOMMENDED BY HEALTH CARE STAFF?

- Yes
- No

ON AVERAGE, APPROXIMATELY HOW MUCH DO YOU SPEND ON NUTRITIONAL SUPPLEMENTS PER WEEK?

TIME LOSS FOR GUARDIANS OR CARERS
• NOT TO BE FILLED IF THE PATIENT IS UNDER 18 YEARS – FOR CHILDREN, ALL QUESTIONS CONCERNING COSTS, TIME SPENT, INCOME, AND INCOME LOSS IN SECTIONS IV AND V CONCERN COST FOR THE GUARDIAN.
• NOTE: OUT-OF-POCKET COSTS OF TRANSPORT, FOOD, ACCOMMODATION FOR GUARDIAN SHOULD BE INCLUDED IN QUESTIONS ON PART V (TABLES).

DID SOMEBODY IN YOUR HOUSEHOLD ACCOMPANY YOU FOR YOUR LAST DOT VISIT?

- Yes
- No

HOW MANY PEOPLE?

DID SOMEBODY IN YOUR HOUSEHOLD ACCOMPANY YOU FOR YOUR LAST VISIT TO PICK UP DRUGS (OR DID THEY PICK UP DRUGS FOR YOU)?

- Yes
- No

HOW MANY PEOPLE?

DID SOMEBODY IN YOUR HOUSEHOLD ACCOMPANY YOU FOR YOUR LAST MEDICAL FOLLOW-UP VISIT?

- Yes
- No

HOW MANY PEOPLE?

DID SOMEBODY IN YOUR HOUSEHOLD ACCOMPANY YOU FOR YOUR LAST HOSPITALISATION?

- Yes
- No

HOW MANY PEOPLE?

HOW MANY PERSONS LOST INCOME DURING THAT TIME?

HEALTH INSURANCE SCHEME

DO YOU HAVE HEALTH INSURANCE?

- Yes
- No

WHAT TYPE?

- Private health insurance
- Other

SPECIFY OTHER.

EDUCATION

WHAT IS YOUR (THE PATIENT'S) EDUCATION LEVEL?

- ◯ Not yet started school
- ◯ Not attended school
- ◯ Should be in school but not attending
- ◯ Pre-school
- ◯ Primary school
- ◯ Secondary school
- ◯ University
- ◯ Vocational
- ◯ Other

SPECIFY OTHER.

WHAT IS THE EDUCATION LEVEL OF PRIMARY INCOME EARNER IN THE HOUSEHOLD (IF DIFFERENT FROM PATIENT)?

- ◯ Not yet started school
- ◯ Not attended school
- ◯ Should be in school but not attending
- ◯ Pre-school
- ◯ Primary school
- ◯ Secondary school
- ◯ University
- ◯ Vocational
- ◯ Other

SPECIFY OTHER.

WHAT IS YOUR CURRENT EMPLOYMENT STATUS?

- Employed/has job
- Doing housework
- Student
- Retired
- Longterm disabled
- Unemployed
- Other

SPECIFY OTHER.

IF YOU HAVE A JOB, WHAT IS YOUR JOB?

- Professional/technical/managerial
- Clerical
- Sales and services
- Skilled manual
- Unskilled manual
- Farmer
- Self employed
- Other

SPECIFY OTHER.

WHAT WAS YOUR EMPLOYMENT STATUS BEFORE YOU CONTRACTED TB?

- Employed/has job
- Doing housework
- Student
- Retired
- Longterm disabled
- Unemployed
- Other

SPECIFY OTHER.

IF YOU HAD A JOB BEFORE YOU CONTRACTED TB, WHAT WAS YOUR JOB?

- Professional/technical/managerial
- Clerical
- Sales and services
- Skilled manual
- Unskilled manual
- Farmer
- Self employed
- Other

SPECIFY OTHER.

ASSET INDEX

WHAT IS YOUR USUAL MAIN SOURCE OF DRINKING WATER?

- Piped water
- Tube well or borehole or protected dug well
- Unprotected Dug well
- Water from spring
- Rainwater
- Surface water
- Piped or Bottled
- Tanker truck
- Bottled water
- Other

SPECIFY OTHER.

WHAT KIND OF TOILET FACILITIES DO YOU HAVE?

- Flush toilet
- Pit latrine
- Composting toilet
- Hanging toilet (including over the sea)
- No toilet/use the bush/beach
- Other

SPECIFY OTHER.

DOES YOUR HOUSEHOLD HAVE:

ELECTRICITY
- Yes
- No

SOLAR POWER
- Yes
- No

A RADIO
- Yes
- No

A TELEVISION
- Yes
- No

A MOBILE PHONE
- Yes
- No

A REFRIGERATOR
- Yes
- No

A COMPUTER
- Yes
- No

A BICYCLE
- Yes
- No

A MOTORBIKE
- Yes
- No

A CAR
- Yes
- No

INCOME (REPORTED) BEFORE CONTRACTING TB

WERE YOU THE PERSON WHO EARNED THE HIGHEST INCOME IN YOUR HOUSEHOLD BEFORE YOU CONTRACTED TB?

- Yes
- No
- Equal contributor
- Not an income earner

HOW WERE YOU USUALLY PAID (BEFORE YOU CONTRACTED TB)?

- [ ] Cash
- [ ] In-kind
- [ ] Unpaid
- [ ] Bank transferred salary
- [ ] Other

SPECIFY OTHER.

HOW MUCH DO YOU ESTIMATE YOUR AVERAGE INCOME, AFTER TAX, PER MONTH WAS BEFORE YOU CONTRACTED TB?

HOW MUCH DO YOU ESTIMATE WAS THE AVERAGE MONTHLY INCOME (AFTER TAX) OF YOUR HOUSEHOLD BEFORE YOU CONTRACTED TB?

HOW MANY HOURS A WEEK WERE YOU WORKING BEFORE YOU CONTRACTED TB?

INCOME CHANGES AND SOCIAL CONSEQUENCES

HOW MUCH DO YOU ESTIMATE IS YOUR AVERAGE INCOME, AFTER TAX, PER MONTH NOW?

HOW MUCH DO YOU ESTIMATE IS THE AVERAGE INCOME AFTER TAX OF YOUR HOUSEHOLD PER MONTH NOW?

HOW MANY HOURS A WEEK ARE YOU WORKING NOW?

HOW MANY HOURS PER WEEK WERE YOU WORKING IN THE INTENSIVE PHASE?

APPROXIMATELY HOW MANY WORKING DAYS OF INCOME HAVE YOU LOST DUE TO YOUR TB ILLNESS OVERALL?

DID YOU OR YOUR HOUSEHOLD RECEIVE ANY SOCIAL WELFARE PAYMENT AFTER YOU WERE DIAGNOSED WITH TB?

- ( ) Yes
- ( ) No

WHAT TYPE OF SOCIAL WELFARE PAYMENT?

- [ ] Financial assistance from member of Parliament
- [ ] Financial assistance from NGO
- [ ] Paid sick leave
- [ ] Other

SPECIFY OTHER.

IF YOU DID RECEIVE SOCIAL WELFARE PAYMENTS, HOW MUCH DID YOU RECEIVE?

DO YOU CURRENTLY RECEIVE VOUCHERS OR GOODS IN KIND TO COPE WITH TB ILLNESS?

- ○ Yes
- ○ No

IF YOU DID RECEIVE VOUCHERS OR GOODS, WHAT TYPE OF VOUCHERS WHERE THEY?

- [ ] Travel voucher
- [ ] Food support
- [ ] Other enablers

FROM WHOM DO YOU RECEIVE THE VOUCHER/ GOODS

- [ ] Government
- [ ] NGO
- [ ] Employer
- [ ] Private donation
- [ ] Other

SPECIFY OTHER.

HOW MANY PEOPLE USUALLY LIVE IN YOUR HOUSEHOLD?

HOW MANY PEOPLE (ADULTS AND CHILDREN) REGULARLY SLEEP IN YOUR HOUSE (INCLUDING PATIENT)?

HOW MANY ROOMS ARE THERE IN THE HOUSE EXCLUDING THE BATHROOM?

BESIDES YOURSELF, DOES ANYONE ELSE OF YOUR HOUSEHOLD RECEIVE TREATMENT FOR TB, NOW OR DURING THE LAST YEAR?

- Yes
- No

HOW MANY?

HAS THE TB ILLNESS (INCLUDING OTHER HOUSEHOLD MEMBERS TREATED IN THE LAST YEAR) AFFECTED YOUR SOCIAL OR PRIVATE LIFE IN ANY WAY?

- No
- Food insecurity
- Divorce or separated from spouse/partner
- Loss of Job
- Interrupted schooling
- Social exclusion

Coping

COPING

DID YOU OR YOUR HOUSEHOLD USE ANY SAVINGS (CASH OR BANK DEPOSITS) TO COVER COSTS DUE TO THE TB ILLNESS?

- Yes
- No

HOW MUCH SAVINGS DID YOU USE BEFORE TB TREATMENT STARTED?

HOW MUCH SAVINGS DID YOU USE IN THE INTENSIVE PHASE?

HOW MUCH SAVINGS DID YOU USE IN THE CONTINUATION PHASE?

HOW MUCH SAVINGS DID YOU USE IN TOTAL (ONLY IF CANNOT DISAGGREGATE)?

DID YOU BORROW ANY MONEY TO COVER COSTS DUE TO THE TB ILLNESS? (INCLUDING MORTGAGING ANY ASSETS)

- Yes
- No

HOW MUCH DID YOU BORROW BEFORE TB TREATMENT STARTED?

HOW MUCH DID YOU BORROW IN THE INTENSIVE PHASE?

HOW MUCH DID YOU BORROW IN THE CONTINUATION PHASE?

HOW MUCH DID YOU BORROW IN TOTAL (ONLY IF CANNOT DISAGGREGATE)?

FROM WHOM DID YOU BORROW?

- Family
- Neighbours/Friends
- Private Bank
- Cooperative/ Societies
- Colleagues (from work)
- Unofficial lender ("Black market")
- Other

SPECIFY OTHER.

ARE YOU EXPECTED TO PAY THE LOAN(S) BACK?

- Yes
- No

HAVE YOU STARTED PAYING BACK THE LOAN?

- Yes
- No

WHEN DID YOU START?

- ○ Before treatment started
- ○ During the Intensive treatment phase
- ○ During the continuation phase

WHAT IS THE MONTHLY REPAYMENT ON THE LOAN, INCLUDING INTEREST?

DO YOU HAVE ANY CURRENT OUTSTANDING LOANS THAT YOU ARE NOT ABLE TO PAY BECAUSE YOU ARE SICK?

- ○ Yes
- ○ No

HAVE YOU SOLD ANY OF YOUR PROPERTY TO FINANCE THE COST OF THE TB ILLNESS (INCLUDING PAWNING)?

- ○ Yes
- ○ No

WHAT DID YOU SELL?

- ☐ Land
- ☐ Livestock
- ☐ Transport/vehicle
- ☐ Household item
- ☐ Farm produce
- ☐ Gold/Jewelry
- ☐ Other

SPECIFY OTHER.

WHEN DID YOU SELL PROPERTY?

- ☐ Before treatment started
- ☐ During the Intensive treatment phase
- ☐ During the continuation phase

HOW MUCH MONEY DID YOU RECEIVE FROM THE SALE OF ALL ITEMS OF YOUR PROPERTY BEFORE TB TREATMENT STARTED?

HOW MUCH MONEY DID YOU RECEIVE FROM THE SALE OF ALL ITEMS OF YOUR PROPERTY IN THE INTENSIVE PHASE?

HOW MUCH MONEY DID YOU RECEIVE FROM THE SALE OF ALL ITEMS OF YOUR PROPERTY IN THE CONTINUATION PHASE?

HOW MUCH MONEY DID YOU RECEIVE FROM SALE OF ALL ITEMS OF YOUR PROPERTY IN TOTAL (IF CANNOT DISAGGREGATE)?

THE ASSETS THAT YOU SOLD, WERE THEY PREVIOUSLY SUPPORTING THE FAMILY INCOME (OR EXPENDITURE)?

- Yes
- No

INDICATE MONTHLY INCOME PREVIOUSLY GENERATED BY THE ASSETS

WHAT IS THE ESTIMATED MARKET VALUE OF THE PROPERTY YOU SOLD?

DID ANYONE IN YOUR HOUSEHOLD DROP OUT OF SCHOOL OR INTERRUPT SCHOOLING TO ASSIST THE HOUSEHOLD AS A CONSEQUENCE OF YOUR TB ILLNESS?

- Yes
- No

HOW MANY PEOPLE?

1

WHAT IS THE AGE OF THIS CHILD WHO DROPPED OUT?

WHAT IS THE SEX OF THIS CHILD WHO DROPPED OUT?

- Male
- Female

FOR HOW LONG DID THEY DROP OUT FOR (IN WEEKS)?

CLICK "+" TO ADDITIONAL CHILD WHO DROPPED OUT

ON A SCALE OF 1 TO 5, IN WHICH 1 IS NO IMPACT AND 5 IS VERY SERIOUS IMPACT, TO WHAT EXTENT HAS THE TB ILLNESS AFFECTED THE FAMILY FINANCIALLY?

- ○ 1. No impact
- ○ 2. Little impact
- ○ 3. Moderate impact
- ○ 4. Serious impact
- ○ 5. Very serious impact

COMMENTS BY INTERVIEWER
