# Supplementary material for: Economic evaluation of patient costs associated with tuberculosis diagnosis and care in Solomon Islands
Source: BMC Public Health. 2021 Oct 23;21:1928. doi: 10.1186/s12889-021-11938-8 (PMC8542301; doi:10.1186/s12889-021-11938-8)
Supplement: Supplementary file 2 — Additional file 2: Table S1. Household income by type of income and by decile in the Solomon Islands Household Income and Expenditure Survey, 2013 (in Solomon Islands dollars). Table S2. Mean number of facility visits and diagnostic delay for participants in the Solomon Islands national tuberculosis patient cost survey: 2017–2019. Table S3. Tuberculosis affected households classified as facing catastrophic costs by different thresholds, from the Solomon Islands national tuberculosis patient cost survey: 2017–2019. Table S4. Re-classification of tuberculosis-affected households into income decile based on the 2013 Solomon Islands Household Income and Expenditure Survey. Table S5. Estimated median total costs (USD) incurred with adjusted household income from the Solomon Islands national tuberculosis patient cost survey: 2017–2019. [file 12889_2021_11938_MOESM2_ESM.docx]

**Supplementary tables**

Table 1. Household income by type of income and by decile in the Solomon Islands Household Income and Expenditure Survey, 2013 (in Solomon Islands dollars)

| **Decile group** | | **Cash-based income** | | **Home  produce** | | **Imputed Rent** | | **Income in kind** | | | **Total** | |
| --- | --- | --- | --- | --- | --- | --- | --- | --- | --- | --- | --- | --- |
|  |  | **n** | **%** | **n** | **%** | **n** | **%** | **n** | **%** | **n** | | **%** |
| Decile | 1 | 28,154 | **23.7%** | 55,149 | 46.4% | 35,277 | 29.7% | 215 | 0.2% | 118,794 | | 100.0% |
| Decile | 2 | 63,383 | **29.4%** | 105,638 | 49.1% | 45,669 | 21.2% | 651 | 0.3% | 215,342 | | 100.0% |
| Decile | 3 | 88,999 | **31.8%** | 134,920 | 48.1% | 55,894 | 19.9% | 465 | 0.2% | 280,278 | | 100.0% |
| Decile | 4 | 127,748 | **37.3%** | 146,159 | 42.7% | 67,527 | 19.7% | 758 | 0.2% | 342,192 | | 100.0% |
| Decile | 5 | 172,905 | **42.3%** | 153,686 | 37.6% | 80,309 | 19.6% | 1,859 | 0.5% | 408,758 | | 100.0% |
| Decile | 6 | 229,362 | **46.5%** | 178,118 | 36.1% | 82,994 | 16.8% | 2,755 | 0.6% | 493,230 | | 100.0% |
| Decile | 7 | 306,259 | **50.9%** | 180,641 | 30.0% | 110,138 | 18.3% | 4,690 | 0.8% | 601,727 | | 100.0% |
| Decile | 8 | 411,845 | **55.1%** | 186,822 | 25.0% | 133,211 | 17.8% | 15,455 | 2.1% | 747,333 | | 100.0% |
| Decile | 9 | 627,263 | **62.3%** | 192,376 | 19.1% | 158,721 | 15.8% | 28,044 | 2.8% | 1,006,403 | | 100.0% |
| Decile | 10 | 2,496,440 | **81.4%** | 117,019 | 3.8% | 312,931 | 10.2% | 141,484 | 4.6% | 3,067,875 | | 100.0% |

Table 2. Mean number of facility visits and diagnostic delay for participants in the Solomon Islands national tuberculosis patient cost survey: 2017-2019

| **Frequency of visits, drug pick up and diagnostic delay** | **Extra-pulmonary TB**  **N=36**  **Mean (95% CI)** | **Pulmonary TB**  **N=147**  **Mean (95 % CI)** | **All TB patients**  **N=183**  **Mean (95% CI)** | **p value** |
| --- | --- | --- | --- | --- |
| **Number of facility visits** | | | | |
| Before diagnosis | 3.1 (1.7, 4.4) | 2.9 (2.2, 3.7) | 2.9 (2.3, 3.6) | 0.830 |
| DOT | 14.3 (-0.1, 28.6) | 13.2 (8.1, 18.2) | 13.4 (8.6, 18.1) | 0.860 |
| Drug pick-up | 18.8 (14.2, 23.5) | 13.4 (11.5, 15.3) | 14.3 (12.5, 16.1) | 0.025 |
| Follow-up | 4.4 (1.0, 7.7) | 3.4 (2.2, 4.6) | 3.6 (2.5, 4.8) | 0.492 |
| Total | 18.6 (11.5, 25.7) | 16.5 (13.1, 19.9) | 16.9 (13.9, 20.0) | 0.589 |
| **Number of weeks before diagnosis** | 18.0 (8.5, 27.5) | 11.2 (5.9, 16.4) | 12.7 (8.2, 17.3) | 0.210 |
| **Diagnosis delay*** | | | | |
| No | 8 (42.1%) | 31 (48.4%) | 39 (47.0%) | 0.627 |
| Yes | 11 (57.9%) | 33 (51.6%) | 44 (53.0%) |  |
| **Frequency of drug pickup** | | | | |
| Every month | 2 (15.4%) | 11 (16.2%) | 13 (16.0%) | 0.216 |
| Every two weeks | 2 (15.4%) | 29 (42.6%) | 31 (38.3%) |  |
| Every week | 9 (69.2%) | 27 (39.7%) | 36 (44.4%) |  |
| Other | 0 (0.0%) | 1 (1.5%) | 1 (1.2%) |  |

Abbreviations: CI=Confidence interval; TB=Tuberculosis

*Diagnostic delay was defined as an interval greater than four weeks between symptom onset and diagnosis of tuberculosis.

Table 3. Tuberculosis affected households classified as facing catastrophic costs by different thresholds, from the Solomon Islands national tuberculosis patient cost survey: 2017-2019

| **Threshold as a percentage of annual household income** | **Tuberculosis affected households facing catastrophic costs**  **n (%)** |
| --- | --- |
| **Direct and indirect costs combined** | |
| 10% | 174 (95.1) |
| 20% | 169 (92.3) |
| 30% | 165 (90.2) |
| 40% | 161 (88.0) |
| 50% | 157 (85.8) |
| 60% | 143 (78.1) |
| **Direct costs only** | |
| 10% | 158 (86.3) |
| 20% | 149 (81.4) |
| 30% | 136 (74.3) |
| 40% | 127 (69.4) |
| 50% | 119 (65.0) |
| 60% | 110 (60.7) |

Table 4. Re-classification of tuberculosis-affected households into income decile based on the 2013 Solomon Islands Household Income and Expenditure Survey

| **Income classification based on the Solomon Islands HIES 2013** | **Extra-pulmonary TB**  **N=36**  **n (%)** | **Pulmonary TB**  **N=147**  **n (%)** | **All TB patients**  **N=183**  **n (%)** | **p value** |
| --- | --- | --- | --- | --- |
| Decile 1 (Poorest) | 36 (100.0%) | 135 (91.8%) | 171 (93.4%) | 0.370 |
| Decile 2 | 0 (0.0%) | 8 (5.4%) | 8 (4.4%) |  |
| Decile 3 | 0 (0.0%) | 3 (2.0%) | 3 (1.6%) |  |
| Decile 4 | 0 (0.0%) | 1 (0.7%) | 1 (0.5%) |  |
| Decile 5 | 0 (0.0%) | 0 (0.0%) | 0 (0.0%) |  |
| Decile 6 | 0 (0.0%) | 0 (0.0%) | 0 (0.0%) |  |
| Decile 7 | 0 (0.0%) | 0 (0.0%) | 0 (0.0%) |  |
| Decile 8 | 0 (0.0%) | 0 (0.0%) | 0 (0.0%) |  |
| Decile 9 | 0 (0.0%) | 0 (0.0%) | 0 (0.0%) |  |
| Decile 10 (Wealthiest) | 0 (0.0%) | 0 (0.0%) | 0 (0.0%) |  |

Abbreviations: HIES=Household Income and Expenditure Survey; TB=Tuberculosis

Table 5. Estimated median total costs (USD) incurred with adjusted household income from the Solomon Islands national tuberculosis patient cost survey: 2017-2019

| **Cost category** | **Extra-pulmonary TB**  **N=36**  **Median (IQR) in USD** | **Pulmonary TB**  **N=147**  **Median (IQR) in USD** | **All patients**  **N=183**  **Median (IQR) in USD** | **p value** |
| --- | --- | --- | --- | --- |
| **Before TB diagnosis** | | | | |
| Direct medical | 0 (0, 0) | 0 (0, 0) | 0 (0, 0) | **0.033** |
| Direct non-medical | 6 (5, 10) | 6 (4, 6) | 6 (4, 6) | 0.577 |
| ***Total direct*** | 6 (5, 10) | 6 (4, 6) | 6 (5, 6) | 0.706 |
| **During TB treatment: direct medical** | | | | |
| Drug pickup | 0 (0, 0) | 0 (0, 0) | 0 (0, 0) | 0.776 |
| DOT | 0 (0, 0) | 0 (0, 0) | 0 (0, 0) | 0.675 |
| Follow up | 0 (0, 0) | 0 (0, 0) | 0 (0, 0) | 0.843 |
| Hospitalization | 58 (0, 75) | 33 (0, 62) | 36 (0, 68) | 0.181 |
| ***Total direct medical*** | 58 (0, 76) | 36 (0, 69) | 37 (0, 71) | 0.260 |
| **During TB treatment: direct non-medical** | | | | |
| Transportation | 76 (65, 167) | 30 (10, 106) | 39 (12, 123) | **<0.001** |
| Accommodation | 58 (0, 72) | 31 (0, 59) | 32 (0, 65) | 0.109 |
| Food during visits | 75 (59, 130) | 40 (9, 128) | 59 (11, 128) | 0.027 |
| Nutritional supplement | 320 (0, 880) | 64 (0, 427) | 85 (0, 427) | 0.097 |
| ***Total direct non-medical*** | 501 (212, 1,323) | 395 (122, 826) | 435 (146, 961) | **0.046** |
| **Entire TB episode** | | | | |
| Direct medical | 58 (0, 76) | 36 (0, 70) | 38 (0, 73) | 0.292 |
| Direct non-medical | 509 (218, 1,330) | 390 (113, 846) | 435 (141, 961) | **0.042** |
| Total direct | 630 (271, 1,390) | 486 (165, 989) | 494 (189, 1,110) | **0.044** |
| Income loss | 416 (0, 1,090) | 312 (0, 1,403) | 312 (0, 1,247) | 0.481 |
| ***Grand total*** | 1,202 (727, 2,777) | 1,107 (521, 2,241) | 1,193 (532, 2,346) | 0.195 |

Abbreviations: DOT=Directly observed therapy; IQR=Inter quartile range; EPTB=Extra pulmonary TB; NA=Not applicable; PTB=Pulmonary TB; USD: United States Dollars; TB=Tuberculosis
